# A BTB extension and ion-binding domain contribute to the pentameric structure and TFAP2A binding of KCTD1

**DOI:** 10.1016/j.str.2024.07.023

**Published:** 2024-08-26

**Authors:** Daniel M. Pinkas, Joshua C. Bufton, Alice E. Hunt, Charlotte E. Manning, William Richardson, Alex N. Bullock

**Affiliations:** 1 Centre for Medicines Discovery, Nuffield Department of Medicine Research Building, University of Oxford, Roosevelt Drive, Oxford OX3 7FZ, UK

## Abstract

KCTD family proteins typically assemble into cullin-RING E3 ligases. KCTD1 is an atypical member that functions instead as a transcriptional repressor. Mutations in KCTD1 cause developmental abnormalities and kidney fibrosis in scalp-ear-nipple syndrome. Here, we present unexpected mechanistic insights from the structure of human KCTD1. Disease-causing mutation P20S maps to an unrecognized extension of the BTB domain that contributes to both its pentameric structure and TFAP2A binding. The C-terminal domain (CTD) shares its fold and pentameric assembly with the GTP cyclohydrolase I feedback regulatory protein (GFRP) despite lacking discernible sequence similarity. Most surprisingly, the KCTD1 CTD establishes a central channel occupied by alternating sodium and iodide ions that restrict TFAP2A dissociation. The elucidation of the structure redefines the KCTD1 BTB domain fold and identifies an unexpected ion-binding site for future study of KCTD1’s function in the ectoderm, neural crest, and kidney.

## Graphical abstract

**Graphical abstract:**
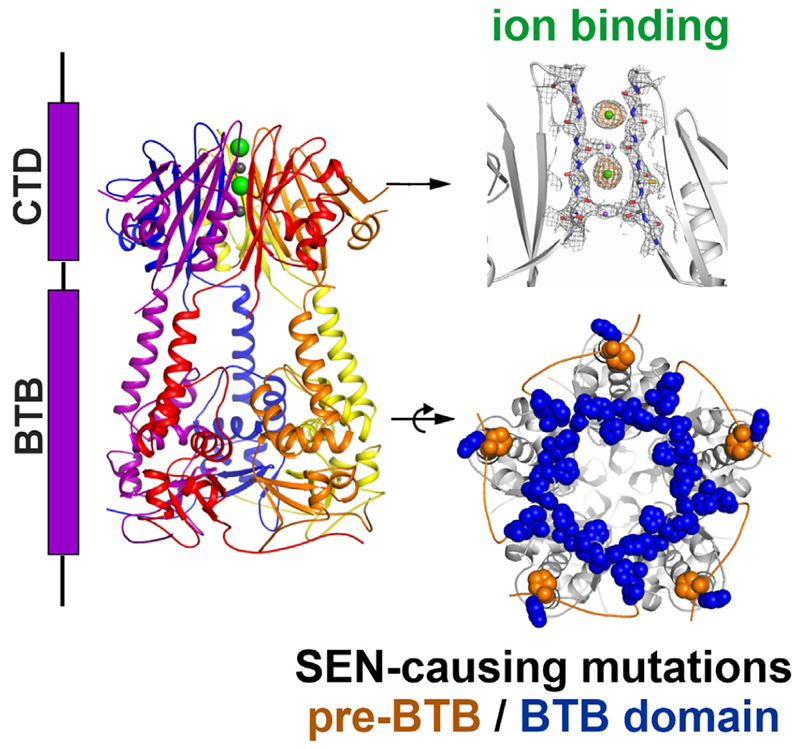

## Introduction

The 25 human members of the KCTD (K^+^ channel tetramerization domain) family form a subgroup of BTB domain-containing proteins that commonly assemble as cullin3-dependent E3 ligases and use a variable C-terminal domain for substrate recognition.^1–4^ Structural studies have been central to their characterization. The prototypical KCTD5 was the first member to have its protein structure elucidated and remains the only family member for which the available structures span both domains.^5–7^ This protein is observed to act as an off-switch for G protein-coupled receptor signaling through the ubiquitin-mediated degradation of G-protein βγ subunits.^6,8,9^ Its BTB domain displays significant structural conservation with the potassium (K^+^) channel tetramerization domain (T1), from which the KCTD family derives its name, but adopts a closed pentameric assembly that binds to cullin3 with 5:5 stoichiometry.^5,7^ The associated pentameric C-terminal domain (CTD) of KCTD5 has a βαββ fold that can similarly assemble as a 5:5 complex with Gβγ subunits, further highlighting the importance of oligomerization and co-operative binding for KCTD family function.^6,7^

Not all KCTD family proteins demonstrate binding to cullin3.^3,4,10^ Structural and biophysical studies of the clade F^1,11^ proteins KCTD8, KCTD12, and KCTD16 have provided insights into their function instead as auxiliary subunits of GABA_B_ receptors. Tethered to GABA_B2_ by their BTB domains, the CTDs of these KCTD proteins bind to Gβγ subunits released from Gα, resulting in the rapid deactivation of downstream GIRK (G protein-gated inwardly rectifying potassium) channel activity.^12–15^ Remarkably, the structure of the 5:5 KCTD12-Gβγ complex reveals an expanded CTD (the H1 domain) that exhibits an entirely distinct protein-protein interface to the equivalent KCTD5 complex.^12^ Indeed, recent work suggests that Gβγ interaction may be a common trait within the different KCTD family subgroups despite their diverged structural mechanisms.^9^

The clade A proteins KCTD1 and KCTD15 form a further distinct, but important subgroup with no binding to cullin3 and no apparent role in G-protein regulation.^3,10^ These proteins instead repress the transactivation domain of the transcription factor TFAP2A (AP-2α).^16,17^ Importantly, disease-causing mutations in KCTD1, KCTD15, and TFAP2A that abrogate this function are associated with craniofacial abnormalities and cutis aplasia.^16,18–21^ Missense substitutions in the BTB domain of KCTD1 cause scalp-ear-nipple (SEN) syndrome, an autosomal dominant condition characterized by facial dysmorphism and ectodermal abnormalities, including sparse hair and the absence of nipples, as well as progressive renal fibrosis.^22,23^ Mutant KCTD1 proteins display reduced TFAP2A binding and thermostability resulting in a propensity to form amyloid-like aggregates and dominant-negative loss of function in mixed models of wild type and mutant.^16,19,20^ Their effects are partly understood from the structures of the truncated BTB domain,^3^ with the notable exception of the P20S mutation, which falls outside the known domains of the KCTD family.

Here, we analyzed the structure, stability, and interactions of full-length KCTD1 to better understand the functionally important N terminus, the spatial arrangement of BTB-CTD domains, and the previously uncharacterized clade A CTD fold. We uncovered unexpected structural features across the length of KCTD1 that contribute to its pentameric assembly and TFAP2A interaction.

## Results

### Structure of full-length KCTD1

**Figure 1:**
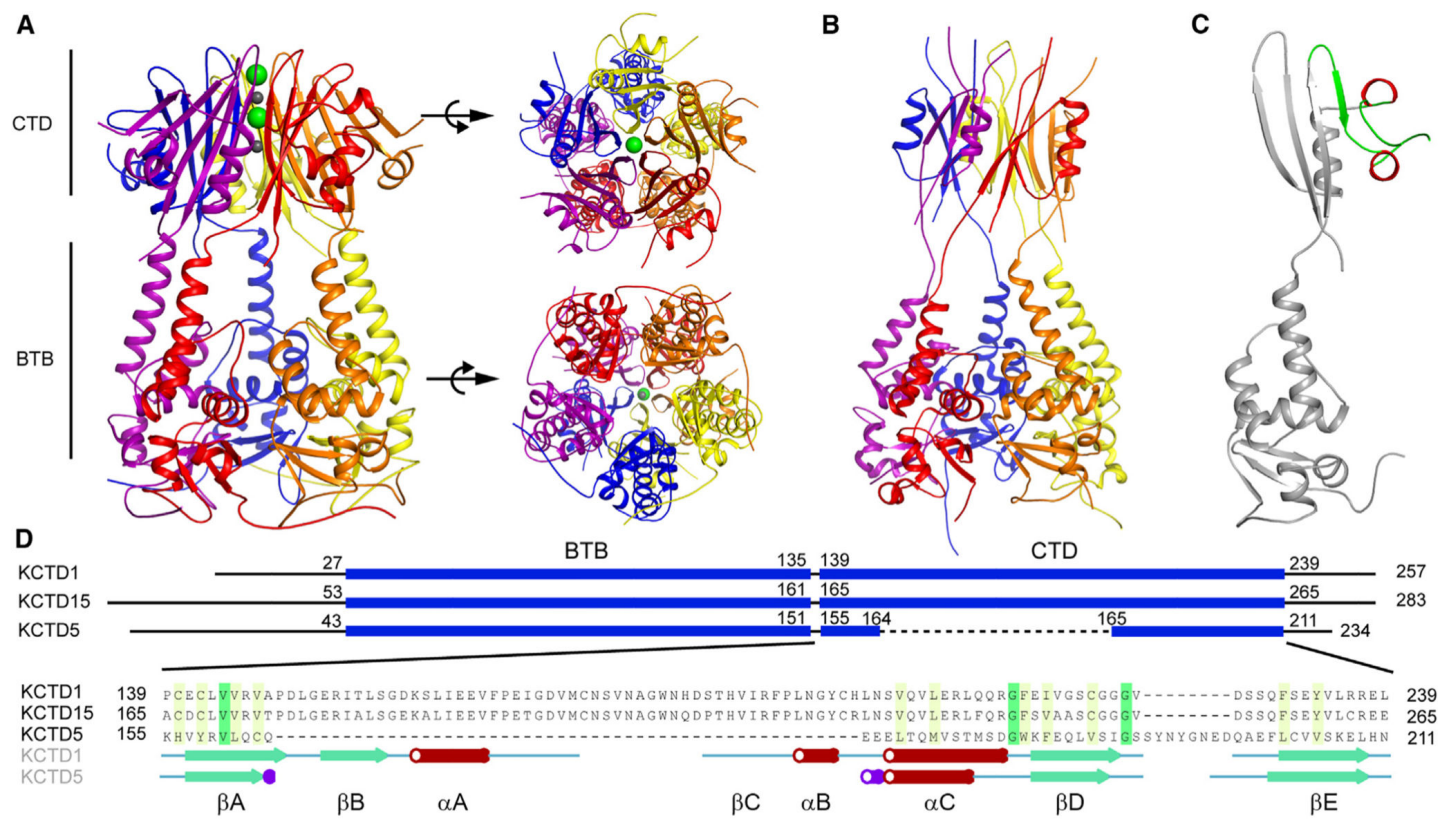
Structure of full-length KCTD1 (A) Ribbon diagram of the pentameric KCTD1 structure. Sodium and iodide ions are shown as gray and green spheres, respectively. (B) Previously determined KCTD5 structure (PDB 3DRY). (C) Single chain of KCTD1. An insertion in the CTD not found in KCTD5 is highlighted in red (helix) and green (β strand and coil). (D) Structure-based alignment of the CTDs of KCTD1, KCTD15, and KCTD5. See also Figure S1.

To understand the structural mechanisms of the multidomain KCTD1 protein, we purified both full-length and truncated forms of the KCTD1 protein and performed crystallization screening. Recombinant human KCTD1 was observed to crystallize in coarse screen conditions containing sodium chloride or sodium iodide. Fine screening revealed that high concentrations of sodium iodide were required for reproducible diffracting crystals. A 2.4 Å structure was initially obtained for KCTD1_ΔN27_ (removing 27 N-terminal residues) and subsequently a 2.7 Å structure of the full-length protein (Figures 1 and S1; Table 1). The structures reveal that KCTD1 forms a closed homopentamer, as found for full-length KCTD5. A short helical linker connects the BTB and CTD domains, which share nearly coaxial C5 symmetry axes. The CTDs are twisted around that axis by a full pentameric subunit with respect to the BTB domains.

Only 3 of the 101 residues in the CTD of KCTD1 are conserved in KCTD5. Yet, remarkably, the domains are found to be structurally related (root-mean-square deviation [RMSD] = 2.2 Å over 50 Cα atoms), with KCTD1 exhibiting a large insertion with respect to the CTD of KCTD5, nearly doubling its size from 6 to 11 kDa. While the KCTD5 CTD displays a relatively “open” solvent-filled central channel, the CTD of KCTD1 exhibits a tightly restricted channel occupied by sodium and iodide ions sequestered from the crystallization solution (Figures 1 and S1).

### An extended BTB domain in KCTD1 is required for TFAP2 binding

**Figure 2:**
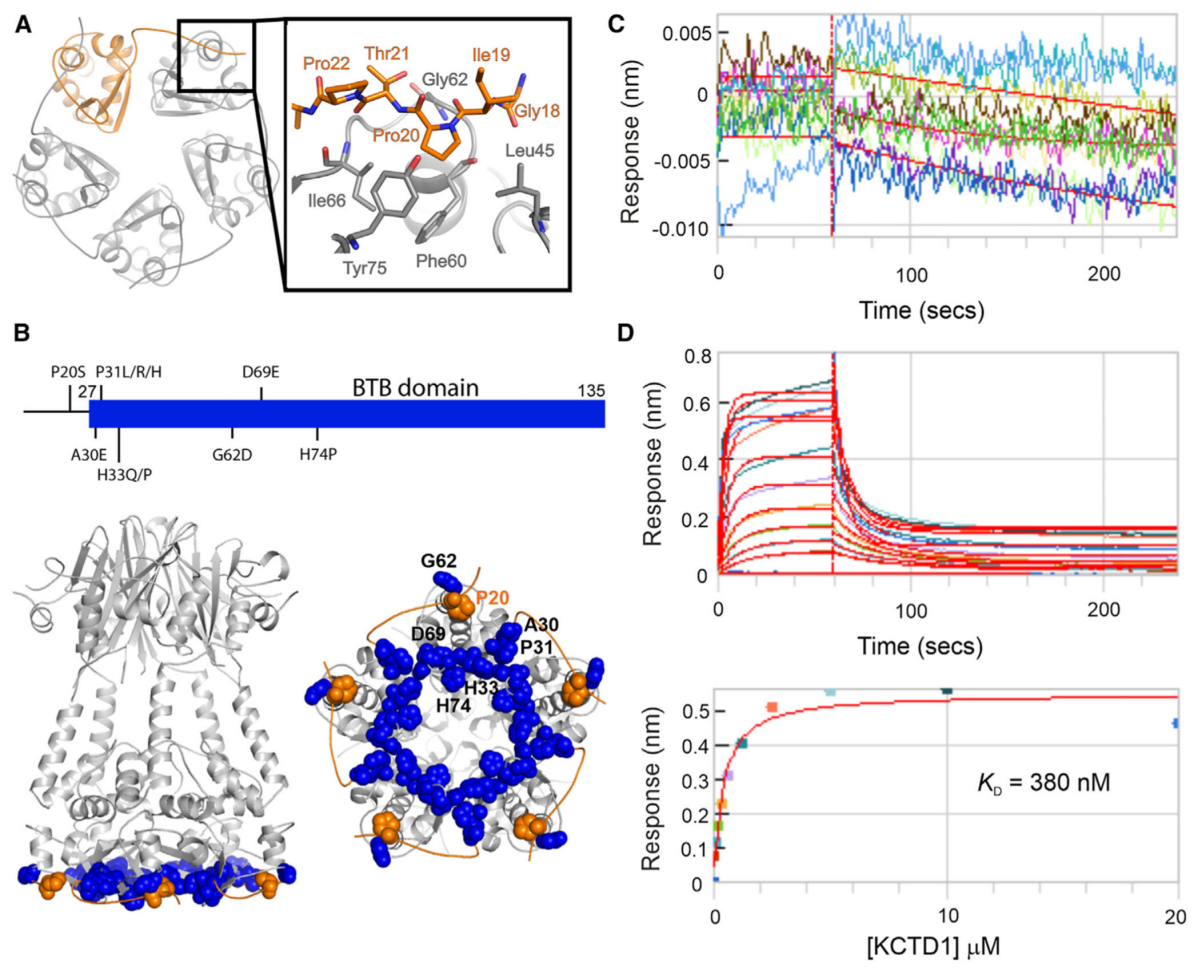
An extended BTB domain in KCTD1 is required for TFAP2 binding (A) Pentameric BTB domain assembly highlighting one chain in orange. Inset shows interactions of the N-terminal BTB extension with the neighboring subunit. (B) Residues associated with SEN-causing mutations are clustered in the BTB domain structure and are shown in spacefill representation colored blue, or orange for Pro20. (C and D) Biolayer interferometry (BLI) measurements. Biotinylated TFAP2A peptide was immobilized on streptavidin-coated sensor tips. Full-length KCTD1 protein was titrated up to 20 μM to assess binding. (C) KCTD1 amino acid (aa) 28–257 shows no binding. (D) Full-length KCTD1 shows dose-dependent binding to TFAP2A with K_D_ = 380 nM by BLI. Protein was buffered in 25 mM HEPES pH 7.5, 150 mM NaCl, 0.05% Tween 20, and 0.5 mM TCEP. See also Figure S4.

A most unexpected and striking feature of the full-length KCTD1 structure was the oligomeric assembly of the BTB domain. Previous KCTD family structures were often truncated to the globular BTB domain as in KCTD1_ΔN27_. The full-length KCTD1 structure reveals a hitherto unrecognized 11-residue N-terminal extension of the BTB domain that drapes across the neighboring BTB domain to establish a ring of intersubunit interactions around the outside of the BTB pentamer (Figure 2A). Importantly, this packing provides a structural explanation for the disease-causing KCTD1 mutations P20S and G62D (and equivalent KCTD15 mutation G88D) that are known to destabilize the proteins and reduce TFAP2 binding (Figures 2A and 2B).^20^ The structure reveals that Pro20 falls within the N-terminal extension and inserts into a hydrophobic pocket in the neighboring BTB to contact residues Leu45, Phe60, Gly62, Ile66, and Tyr75 in this domain. The mutations P20S and G62D would abrogate this hydrophobic packing and affect the backbone conformation. Consistent with this mutation, KCTD1_ΔN27_ lacking the N-terminal extension displayed minimal binding to a TFAP2A peptide derived from the transactivation domain, whereas full-length KCTD1 exhibited submicromolar affinity further highlighting the importance of the N-terminal BTB extension for KCTD1 function (Figures 2C and 2D). The full-length KCTD1 structure was obtained from a crystallization drop containing TFAP2A peptide with the hope to determine its co-structure. However, the resulting electron density maps provided no evidence for bound peptide and so the precise structural basis for its interaction remains to be elucidated.

### Iodide binding to KCTD1 CTD affects protein stability and TFAP2 binding

**Figure 3:**
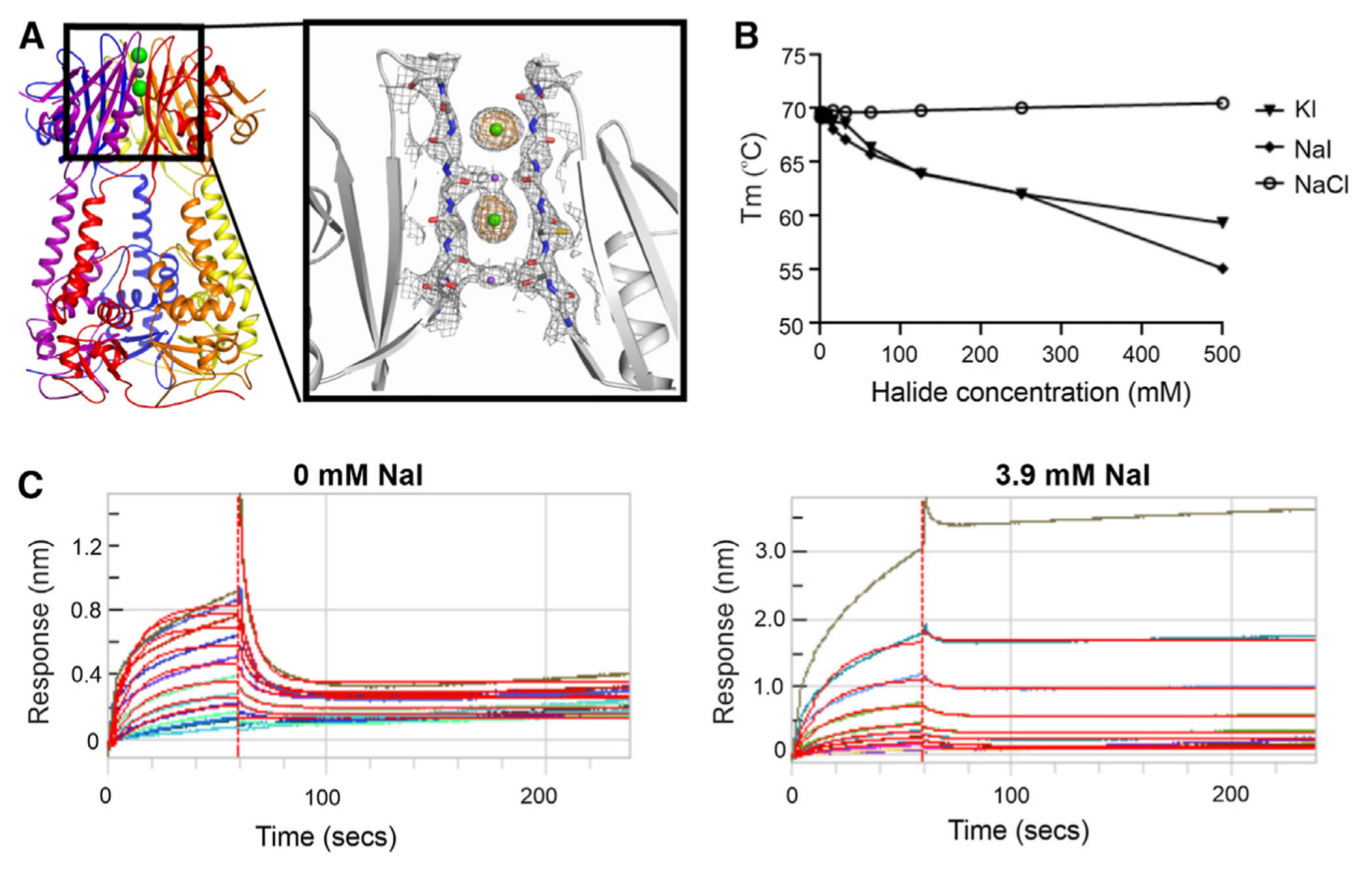
Iodide binding to KCTD1 CTD affects protein stability and TFAP2 binding (A) The KCTD1 pentamer displays an ion-binding channel in the CTD occupied by two sodium and two iodide ions (purple and green spheres, respectively). Inset slice shows 2Fo-Fc electron density map (gray) contoured at 1.0 σ overlaid with iodide-specific anomalous map (brown) contoured at 5.0 σ. (B) Apparent melting temperature of full-length KCTD1 at different halide ion salt concentrations. Protein was buffered in 25 mM HEPES pH 7.5, 0.05% Tween 20 plus indicated halide salts and SYPRO orange dye. Dilutions from 500 mM salt were mixed with NaH_2_PO_4_ pH 7.5 to maintain constant ionic strength. (C) BLI measurements of KCTD1 binding to immobilized TFAP2A peptide. Full-length KCTD1 protein was titrated up to 20 μM to assess binding buffered in 25 mM HEPES pH 7.5, 150 mM NaCl, 440 mM NaH_2_PO_4_ pH 7.5, 0.05% Tween 20, and indicated NaI concentrations. See also Figure S2.

Electron density maps indicated the presence of presumed sodium and iodide ions in the central channel formed by the five CTD βD strands (Figures 3A and S2A). By optimizing the crystallization conditions, we were able to increase the occupancy of two iodides to unity. Their identity was further confirmed by a diffraction dataset collected at 7,000 eV that revealed a peak anomalous signal of 15 σ. Interestingly, the sodium ions were coordinated by the backbone carbonyls and side-chain hydroxyls of a βD Ser-X-Gly-X-Gly motif that was weakly reminiscent of the same groups in the Thr-X-Gly-X-Gly selectivity sequence of the pore loop domain in the Kv channel family (Figure S2B).

We investigated whether the bound ions were relevant to the structure and function of KCTD1. Sodium and potassium iodide salts were found to be destabilizing as shown by a reduction in the apparent melting temperature of the full-length protein (Figures 3B and S2C). This effect was specific to iodide as the melting temperature was unaffected by sodium chloride concentration irrespective of conditions of constant (Figure 3B) or variable ionic strength (Figure S2C). We extended these experiments to the isolated BTB and CTD domains and observed that the destabilization induced by iodide occurred only with the CTD consistent with the ion-binding site, whereas the BTB domain was stabilized (Figure S2C). Finally, we found that even low millimolar concentrations of sodium iodide were sufficient to alter the binding of KCTD1 to TFAP2A and limit the dissociation of the peptide from the bound complex (Figure 3C). These results show that sodium iodide binding to the CTD affects both the stability and ligand interactions of KCTD1.

### KCTD1 CTD shares structural homology with GFRP as well as KCTD5 and KCTD12

**Figure 4:**
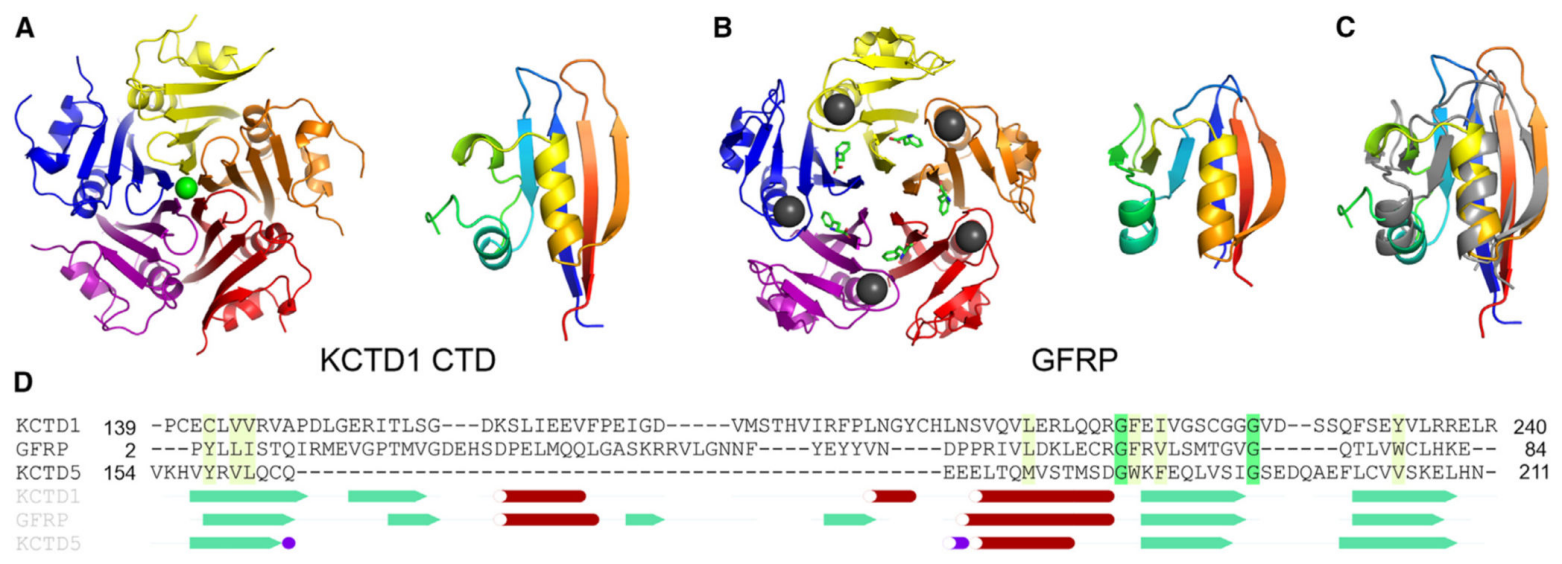
KCTD1 CTD shares structural homology with GFRP (A) KCTD1 CTD pentamer (left) and subunit topology by rainbow colors (right). (B) Human GFRP pentamer (PDB 7AL9, left) and chain topology (right). Bound phenylalanine and potassium ions are shown as green sticks and gray spheres, respectively. (C) Superposition of KCTD1 CTD (rainbow) and GFRP (gray) chains. (D) Structure-based sequence alignment. See also Figure S3.

The CTD structure comprises a five-stranded antiparallel β sheet (βA-βE) interrupted by two α helices (αA and αC) and a 3_10_-helix (αB) (Figures 1C, 1D, and 4A). Prior to structure determination, the KCTD1 CTD showed 80% sequence identity to its paralog KCTD15, but had no other obvious matches by sequence homology, although similarity was subsequently suggested by AlphaFold.^11,24^ The structure helps to define a 40-residue insertion (βB-αA-βC-αB) relative to KCTD5 that enables its structural conservation to be recognized (Figures 1C and 1D). In the pentameric assembly, these structural elements lie on the outermost face of the CTD fold, where they potentially form a binding interface for protein partners, as observed for the prototypical KCTD5.^6,7^

A search for structural homologs of the KCTD1 CTD using the DALI server^25^ identified GTP cyclohydrolase I feedback regulatory protein (GFRP) as an unexpected match outside of the KCTD family (RMSD = 2.3 Å over 83 Cα atoms, Figures 4B and 4C), a protein for which there is virtually no sequence similarity (Figure 4D). Moreover, the GFRP protein assembles into a closed pentameric complex nearly identical to that of the CTD of KCTD1 (Figures 4A and 4B). To our surprise, the KCTD12 CTD was another close match (RMSD = 2.2 Å over 83 Cα atoms) revealing far wider conservation within the KCTD family than previously thought (Figure S3). It should be noted, however, that the GFRP, KCTD5, and KCTD12 structures all show “open” solvent-filled central channels in contrast to the tightly restricted ion-bound channel of KCTD1. The clade F proteins KCTD8/12/16 are also distinguished from other KCTD proteins by having large flexible linkers between their BTB and CTD domains (Figure S3).

Overall, the elucidation of the full-length KCTD1 structure uncovers important unanticipated features of the BTB and CTD domains that contribute to its oligomeric assembly and protein-ligand interactions.

## Discussion

This work unveils unanticipated features of the KCTD1 pentamer, as well as general lessons for the KCTD family. The BTB domain is the most characterized region of the KCTDs but is habitually studied using truncated expression constructs. The identified N-terminal extension of the KCTD1 BTB domain presents a striking parallel to the β1 strand of the dimeric BTB protein class. In dimeric BTBs, the N-terminal β1 strand establishes domain-swap interactions that stabilize the dimer interface,^26^ as well as a binding surface for transcriptional repressors.^27^ In an analogous arrangement, the N-terminal region of KCTD1 forms cyclic domain-swap interactions to stabilize the BTB pentamer, as well as a critical region for the binding of TFAP2A. This explains how the N-terminal P20S mutation identified in SEN syndrome disrupts both the thermostability of KCTD1 and its TFAP2A interaction.^20^ We anticipate that this functionally important BTB extension will be conserved in other KCTD family members, such as KCTD15 (see Figure S4), and advise that future studies incorporate it at construct design.

The BTB and CTD domains are tethered by a helix that may allow functional interplay between the two pentameric rings, as evidenced by E3 ligases such as KCTD5.^6,7^ We note that the CTD of KCTD5 displays a relatively “open” solvent-filled central channel, whereas the channel of KCTD1 is restricted to bind to alternating sodium and iodide ions, the most unusual feature of the structure. Similar to the pre-BTB region, ion concentration was found to modulate the TFAP2A interaction suggestive of potential BTB-CTD cross-talk. Despite co-crystallization, we were unable to identify a bound TFAP2A peptide in our structure to shed further insight into this relationship. This interaction may be defined in future by cryoelectron microscopy (cryo-EM) studies using the full-length TFAP2A protein. Cryo-EM may also help to establish whether ion binding to the CTD regulates the diameter of the central channel and whether this impacts the interaction of TFAP2A or other potential ligands. Ion binding is noteworthy as the KCTD1/TFAP2A transcriptional axis regulates the reabsorption of salts in the kidney.^23^

The CTD structure also exposes fold similarities between disparate clades of the KCTD family that are otherwise difficult to discern from their sequences alone. Particularly striking is the structural conservation of KCTD1 (clade A) and KCTD12 (clade F), and their similar insertion relative to the CTD of the prototypical KCTD5 (clade E). These observations strengthen the phylogenetic linkage between the prototypical KCTD family members acting as E3 ligase adaptors and those with alternative functions.^1^ The surprising similarity of the CTDs to GFRP also raises the possibility that these proteins may have arisen from a common ancestor. GFRP acts as a metabolic sensor that also depends on its pentameric structure to bind to GTP cyclohydrolase I as an allosteric inhibitor or activator dependent on the availability of cofactors tetrahydrobiopterin or phenylalanine, respectively.^28–30^

A common thread throughout this work is the importance of oligomerization for KCTD1 function, as evidenced by the contributions of the pre-BTB extension, ion-binding CTD, and dominant-negative mutations in SEN syndrome. Our work sheds further light on the structure-function relationship of KCTD proteins and lays a structural foundation for understanding the non-E3 KCTDs.

## Star★Methods

Detailed methods are provided in the online version of this paper and include the following:

- KEY RESOURCES TABLE
- RESOURCE AVAILABILITY
  - Lead contact
  - Materials availability
  - Data and code availability
- EXPERIMENTAL MODEL AND STUDY PARTICIPANT DETAILS
  - Bacterial strains
- METHOD DETAILS
  - Cloning
  - Expression
  - B Purification
  - Crystallisation
  - Diffraction data collection and structure refinement
  - Biolayer interferometry
  - Differential scanning fluorimetry
- QUANTIFICATION AND STATISTICAL ANALYSIS

## Star★Methods

### Key Resources Table

**Table:** 

REAGENT or RESOURCE	SOURCE	IDENTIFIER
Bacterial and virus strains		
E. coli BL21(DE3)R3-pRARE2 cells	Structural Genomics Consortium (SGC)	Addgene #26242
MACH T1 cells	ThermoFisher	C862003
Chemicals, peptides, and recombinant proteins		
Ni Sepharose High Performance histidine-tagged protein purification resin	GE Healthcare	17526802
HiLoad® 16/600 Superdex® 200 pg	Sigma Aldrich	GE28-9893-35
Full length KCTD1 protein (a.a. 1–257)	This paper	N/A
KCTD1ΔN27 protein (a.a. 28–257)	This paper	N/A
KCTD1 BTB domain (a.a. 26–132)	This paper	N/A
KCTD1 CTD domain (a.a. 132–257)	This paper	N/A
TFAP2A peptide (Biotin-NADFQPPYFPPPYQ, Uniprot P05549-1 residues 50–63	This paper	N/A
Deposited data		
Structure of full-length KCTD1 (a.a. 1–257)	This paper	PDB: 9FOI
Structure of KCTD1ΔN27 (a.a. 28–257)	This paper	PDB: 6S4L
Oligonucleotides		
KCTD1 full length Fwd primer (Eurofin Genomics) TACTTCCAATCCATGTCAAGACCTCTGATCACTAGATCCCC	This paper	N/A
KCTD1 full length Rev primer (Eurofin Genomics) TATCCACCTTTACTGTCAGTCCAGAGGCTCTTGCTTTATCCGG	This paper	N/A
KCTD1ΔN27 Fwd primer (Eurofin Genomics) TACTTCCAATCCATGTCCAATGCGCCTGTCCACATTG	This paper	N/A
KCTD1ΔN27 Rev primer (Eurofin Genomics) TATCCACCTTTACTGTCAGTCCAGAGGCTCTTGCTTTATCCGG	This paper	N/A
KCTD1 BTB Fwd primer (Eurofin Genomics) TACTTCCAATCCATGACAAAATCCAATGCGCCTGTCCAC	This paper	N/A
KCTD1 BTB Rev primer (Eurofin Genomics) TATCCACCTTTACTGTCATTCTCTGTCCTGCTTCCATCTTTCC	This paper	N/A
KCTD1 CTD Fwd primer (Eurofin Genomics) TACTTCCAATCCATGGAAACTGGTCGATTTTCAAGGCCC	This paper	N/A
KCTD1 CTD Rev primer (Eurofin Genomics) TATCCACCTTTACTGTCAGTCCAGAGGCTCTTGCTTTATCCGG	This paper	N/A
Recombinant DNA		
pNIC-NHStllT vector	Structural Genomics Consortium (SGC)	N/A
pCDF-LIC vector	Structural Genomics Consortium (SGC)	N/A
pNIC28-Bsa4 vector	Structural Genomics Consortium (SGC)	Addgene Plasmid #26103
pCMVHA1-KCTD1 (cDNA for KCTD1 PCR)	MRC PPU	Plasmid DU42183
Software and algorithms		
PyMOL 2.3.4	The PyMOL Molecular Graphics System, Version 2.3 Schrödinger, LLC	https://pymol.org/2/#download
XDS	XDS Kabsch, 2010^31^	https://xds.mr.mpg.de/ RRID:SCR_015652
AIMLESS	Winn et al., 2011^32^	https://www.ccp4.ac.uk/html/aimless.html RRID:SCR_015747
PHASER	McCoy et al., 2007^33^	https://phenix-online.org/documentation/reference/phaser.html RRID:SCR_014219
COOT	Emsley et al., 2010^34^	https://www2.mrc-lmb.cam.ac.uk/personal/pemsley/coot/RRID:SCR_014222
PHENIX-REFINE	Adams et al., 2010^35^	https://phenix-online.org/RRID:SCR_014224
MolProbity	Chen et al., 2010^36^	http://molprobity.biochem.duke.edu RRID:SCR_014226
GraphPad Prism version 9 for Windows	GraphPad Software, La Jolla California USA	www.graphpad.com
FortéBio Octet® Data Analysis software (V.9.0)	ForteBio	N/A
Other		
Biolayer Interferometry System	ForteBio	Octet® RED384 RRID:SCR_023267
Streptavidin (SA) biosensors	ForteBio	Cat#18-5020

### Resource Availability

#### Lead contact

Further information and requests for resources and reagents should be directed to and will be fulfilled by the lead contact, Alex N Bullock (alex.bullock@cmd.ox.ac.uk).

#### Materials availability

Plasmids generated in this study will be made available on request.

### Experimental Model and Study Participant Details

#### Bacterial strains

DNA constructs were prepared and purified from MACH1 T1 cells (ThermoFisher C862003). All proteins were recombinantly expressed in *Escherichia coli* BL21(DE3)R3-pRARE2 cells. These BL21(DE3) cells were selected for resistance to environmental bacteriophage at the Structural Genomics Consortium (SGC) and transformed with the pRARE2 plasmid purified from ROSETTA *E. coli* cells. Cells were cultured in Luria-Bertani (LB) broth.

### Method Details

#### Cloning

Constructs were prepared using ligation-independent cloning^37^ in MACH1 T1 cells and using PCR from a cDNA for KCTD1 (pCMVHA1-KCTD1; MRC PPU plasmid DU42183). Full-length human KCTD1 (Uniprot Q719H9, residues 1–257) was inserted into the pNIC-NHStIIT vector which provides N-terminal hexahistidine and tandem Streptag II tags followed by a tobacco etch virus (TEV) cleavage site. The didomain construct of KCTD1 containing residues 28–257 (KCTD1_ΔN27_) was cloned into the pCDF-LIC vector which provides an N-terminal hexahistidine tag and TEV cleavage site. The KCTD1 BTB domain (residues 26–132) was similarly cloned into the pCDF-LIC vector, while the KCTD1 CTD domain (residues 132–257) was cloned into the pNIC28-Bsa4 vector, which also provides an N-terminal hexahistidine tag and TEV cleavage site.

#### Expression

Constructs were transformed into BL21(DE3)-R3-pRARE2 cells. Expression was performed in Luria-Bertani (LB) broth supplemented with appropriate antibiotics (kanamycin and chloramphenicol for the pNIC28-Bsa4 and pNIC-NHStIIT vector constructs, and spectinomycin and chloramphenicol for the pCDF-LIC vector constructs). Cultures were grown at 37°C with shaking at 180 RPM to an OD_600_ value of 0.6–0.8 and then cooled to 18°C before induction overnight by addition of 0.3 mM isopropyl β-D-1-thiogalactopyranoside (IPTG). Cells were harvested by centifugation at 5000 g for 10 min.

#### Purification

Cell pellets were resuspended in binding buffer (50 mM HEPES pH 7.5, 500 mM NaCl, 5 mM Imidazole, 5% glycerol) supplemented with Protease Inhibitor Cocktail Set III, EDTA-Free and 0.5 mM tris(2-carboxyethyl)phosphine (TCEP). Cells were then lysed by sonication performed on ice. Lysates were cleared by centrifugation at 4°C in a JA25.5 rotor for 45 min at 36,200 g. Hexahistidine-tagged KCTD1 proteins were purified from the supernatant using nickel-sepharose resin (GE Healthcare). Captured proteins were washed with binding buffer supplemented with 30 mM imidazole and then eluted stepwise with binding buffer supplemented with 100, 150, 250 or 300 mM imidazole. The flow through, wash and elution fractions were analyzed by SDS PAGE to identify KCTD1-containing fractions, which were then pooled and cleaved overnight with TEV protease. A final clean up step was performed using size exclusion chromatography using a HiLoad 16/600 S200 superdex column (Sigma-Aldrich) buffered in 50 mM HEPES pH 7.5, 300 mM NaCl, 5% glycerol, 1 mM TCEP.

#### Crystallisation

Proteins were concentrated to 10 mg/mL using vivaspin 10 kDa MWCO concentrators (GE healthcare). Crystallisation screens were performed by sitting drop vapor diffusion using 3 Lens Crystallisation Plates (SWISSCI, UK) at 4°C and 20°C and explored six different sparse matrix precipitant screens (LFS, JCSG, HCS, HIN, BCS and SaltRx). Three 150 nL sitting droplets per well were prepared by mixing protein and precipitant in ratios of 2:1, 1:1 and 1:2. Didomain KCTD1_ΔN27_ containing residues 28–257 was buffered in 50 mM HEPES pH 7.5, 300 mM NaCl, 5% glycerol, 1 mM TCEP. Diffracting crystals grew at 20°C in drops mixing 50 nL protein with 100 nL of a mother liquor containing 12% PEG3350, 0.1 M bis-tris-propane pH 7.8, 0.2 M sodium iodide, 10% ethylene glycol. Mounted crystals were cryoprotected in acetonitrile and vitrified in liquid nitrogen. Full-length KCTD1 was buffered in 50 mM HEPES pH 7.5, 300 mM NaCl, 200 mM NaI, 0.5 mM TCEP and mixed 1:1 with TFAP2A (AP-2α) peptide (NADFQPPYFPPPYQ, transactivation domain residues 50–63, Uniprot P05549-1). Diffracting crystals grew at 4°C in drops mixing 100 nL protein with 50 nL of a mother liquor containing 0.1 M SPG pH 6.0 (mix ratio 2:7:7 succinate/sodium dihydrogen phosphate/glycine), 60.0% MPD (2-methyl-2,4-pentanediol). Mounted crystals were cryoprotected in mother liquor plus 25% ethylene glycol and vitrified in liquid nitrogen.

#### Diffraction data collection and structure refinement

Diffraction data were collected at 100 K on Diamond Light Source beamline I04 (KCTD1_ΔN27_ didomain) or I24 (KCTD1 full length). Data were indexed and integrated using XDS^31^ and scaled using AIMLESS in the CCP4 suite.^32^ The structures were solved by molecular replacement in PHASER.^33^ The pentameric KCTD1 BTB domain structure PDB 5BXB,^3^ was used as an initial search model for KCTD1_ΔN27_ and this structure was subsequently used as a search model for the full-length KCTD1 structure. Initial electron density maps for KCTD1_ΔN27_ indicated the presence of ions in the central tunnel of the CTD1, consistent with partial occupancy of sodium and iodide sequestered from the crystallization solution. By optimizing the crystallization conditions, we were able to increase the occupancy of two Iodides in the central tunnel to unity. To further confirm the identity of the ions, a dataset was collected at 7000 eV. The Iodides exhibited a peak anomalous signal of 15σ (Figure 3A). Manual model building was performed in COOT^34^ and refinement completed using PHENIX.REFINE.^35^ The refined structures were validated with MolProbity^36^ and the atomic coordinate files deposited in the Protein DataBank. Structure figures were generated using PyMOL.

#### Biolayer interferometry

Biolayer interferometry (BLI) experiments were performed on an Octet RED384 instrument (FortéBio). Experiments were performed in a buffer containing 25 mM HEPES pH 7.5, 150 mM NaCl, 0.05% (v/v) Tween 20, 0.5 mM TCEP unless stated otherwise. Biotinylated TFAP2A (AP-2α) peptide (sequence: Biotin-NADFQPPYFPPPYQ, Uniprot P05549-1 residues 50–63) was immobilised onto streptavidin-coated fiber optic sensor tips, while ‘free’ reference sensors were used without immobilised peptide. Sensor tips were sequentially dipped into their respective protein samples from low to high concentrations for association phases and then into buffer for dissociation phases. Data were analyzed using the FortéBio Data Analysis software.

#### Differential scanning fluorimetry

Purified proteins in the indicated buffers were mixed with SYPRO Orange dye (1:1000 dilution). Samples were heated from 25°C to 95°C in a Mx3005P real-time PCR instrument (Stratagene). Fluorescence was monitored with excitation and emission filters set to 465 and 590 nm, respectively. Data were exported to GraphPad Prism and fitted to the Boltzmann equation to calculate apparent *T*_m_ values as described previously. Full-length and BTB domain constructs were assayed at 2 μM protein concentration, whereas the KCTD1 CTD construct was assayed at 100 μM protein concentration due to low signal.

### Quantification And Statistical Analysis

X-ray crystallography data collection and refinement statistics are summarized in Table 1. BLI data from individual experiments (*n* = 1) were fit using the steady state analysis tool in the FortéBio Data Analysis software as described in STAR methods. DSF data from individual experiments (*n* = 1) were fit to the Boltzmann equation in GraphPad Prism as described in STAR methods.

## Supplementary Material

Supplemental information can be found online at https://doi.org/10.1016/j.str.2024.07.023.

Figures S1-S4

Highlights

## Figures and Tables

**Table 1:** Diffraction data collection and structure refinement statistics

	KCTD1 full length PDB ID 9FOI	KCTD1_ΔN27_ PDB ID 6S4L
Wavelength (Å)	0.9686	1.771
Resolution range (Å)	114.84–2.71 (2.85–2.71)	67.41–2.8 (2.9–2.8)
Space group	P 1 21 1	P 1 21 1
Unit cell a, b, c (Å) α, β, γ (°)	67.558 96.634 115.995 90 98.088 90	67.454 95.87 104.343 90 92.158 90
Total reflections	277,262 (27,857)	234,273 (24,285)
Unique reflections	40,265 (3,965)	31,509 (3,082)
Multiplicity	6.9 (6.9)	7.4 (7.9)
Completeness (%)	99.79 (98.85)	95.88 (94.98)
Mean I/sigma(I)	7.01 (1.1)	7.56 (1.16)
Wilson B-factor	57.05	53.69
R-merge	0.213 (1.910)	0.2596 (2.066)
R-meas	0.251 (2.253)	0.2796 (2.213)
R-pim	0.132 (1.182)	0.1023 (0.7886)
CC1/2	0.993 (0.428)	0.99 (0.529)
CC*	0.998 (0.755)	0.998 (0.832)
Reflections used in refinement	40,308 (3,970)	31,497 (3,082)
Reflections used for R-free	1,954 (186)	1,481 (159)
R-work	0.206 (0.3104)	0.2466 (0.3580)
R-free	0.245 (0.3708)	0.2759 (0.3883)
Number of non-hydrogen atoms	8,500	8,534
macromolecules	8,352	8,468
ligands	85	4
solvent	63	62
Protein residues	1,048	1,060
RMSD (bonds, Å)	0.0046	0.003
RMSD (angles, °)	0.70	0.90
Ramachandran favored (%)	94.26	92.60
Ramachandran allowed (%)	5.74	7.40
Ramachandran outliers (%)	0.00	0.00
Rotamer outliers (%)	0.44	2.39
Clashscore	2.13	6.69
Average B-factor (Å^2^)	64.8	53.90
macromolecules	64.9	54.08
ligands	62.3	53.59
solvent	51.0	30.15

Statistics for the highest-resolution shell are shown in parentheses.

## Data Availability

The coordinates and structure factors for the crystal structures reported in this article have been deposited in the PDB with accession codes 9FOI (full length KCTD1) and 6S4L (KCTD1_ΔN27_) and are publicly available as of the date of publication. These accession numbers are also listed in the key resources table. This paper does not report original code. Any additional information required to reanalyze the data reported in this paper is available from the lead contact upon request.

## Highlights

- Crystal structure of full-length human KCTD1 that forms a pentamer
- The pre-BTB region is essential for TFAP2A binding and makes domain-swap interactions
- C-terminal domain pentamer forms a central channel with bound sodium and iodide ions
- The structure explains how mutations disrupt KCTD1 in scalp-ear-nipple syndrome

## In brief

Pinkas et al. determined the pentameric structure of full-length KCTD1 revealing a pre-BTB extension forming cyclic domain-swap interactions and a C-terminal domain forming a central channel occupied by sodium and iodide ions. Both features appear important for TFAP2A binding. The structure explains how mutations disrupt KCTD1 in scalpear-nipple syndrome.
