# Supplementary material for: A BTB extension and ion-binding domain contribute to the pentameric structure and TFAP2A binding of KCTD1
Source: Structure. Author manuscript; Available in PMC 2026 Mar 25. (PMC7618933; doi:10.1016/j.str.2024.07.023)
Supplement: Figures S1-S4 [file EMS213002-supplement-Figures_S1_S4.pdf]

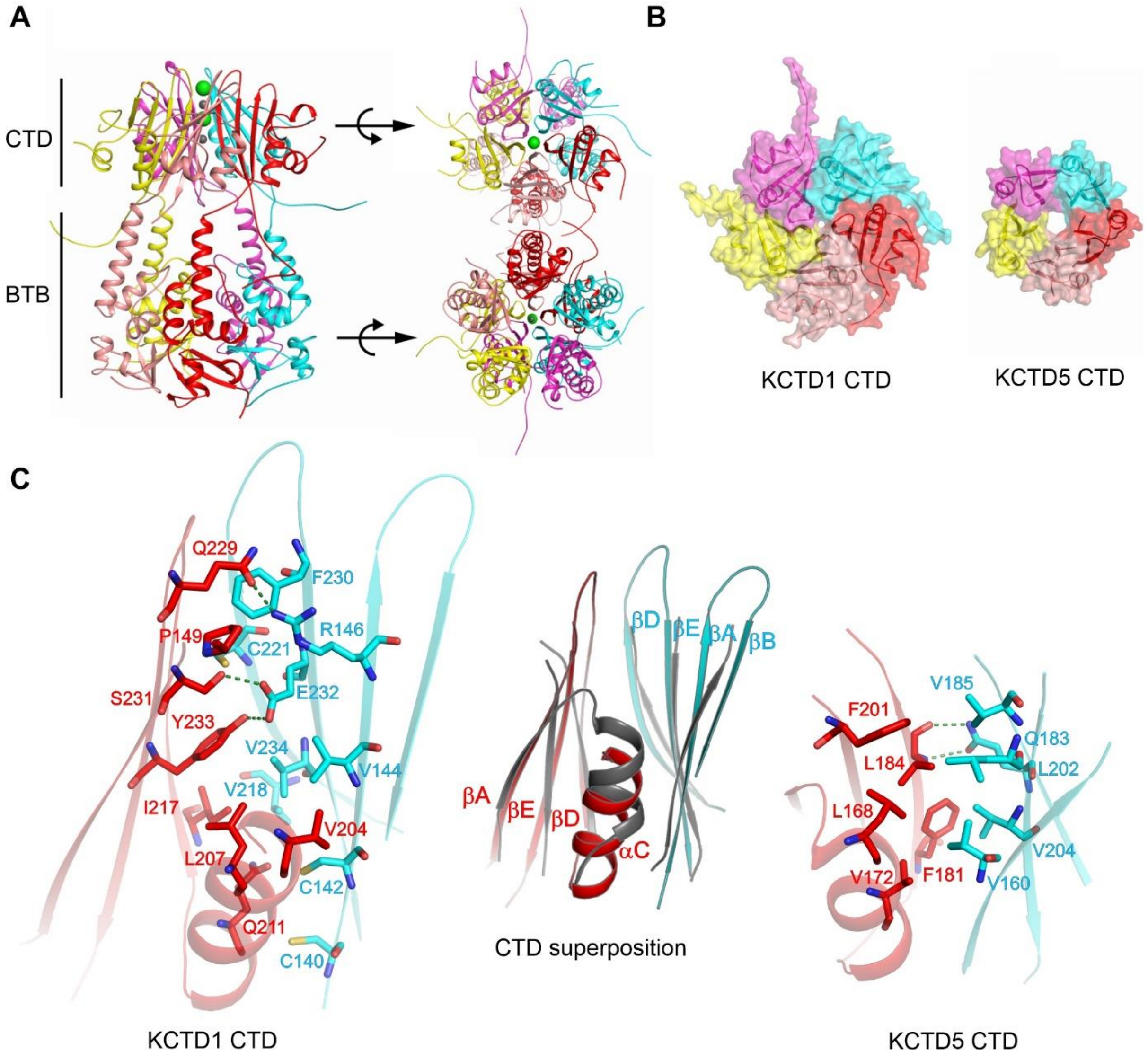

Figure S1. Structure of KCTD1$_{\Delta N27}$, Related to Figure 1

(**A**) Ribbon diagram of the pentameric structure of KCTD1$_{\Delta N27}$. Different protomers are distinguished by different colours. Sodium and iodide ions in the CTD are shown as gray and green spheres, respectively. (**B**) Transparent surface representations of the KCTD1 and KCTD5 CTD pentamers. The central channel in KCTD5 (right) is more open than the ion-bound channel of KCTD1 (left). (**C**) Comparison of intersubunit packing in the CTD domains of KCTD1 and KCTD5 (PDB 3DRX). KCTD1 (left) shows a mix of polar and hydrophobic intersubunit contacts, including several side chain-side chain hydrogen bonds, whereas the KCTD5 intersubunit interface shows a more uniform hydrophobic core (right). Superposition of KCTD1 (cyan/red) and KCTD5 (gray) is not shown to scale (center).

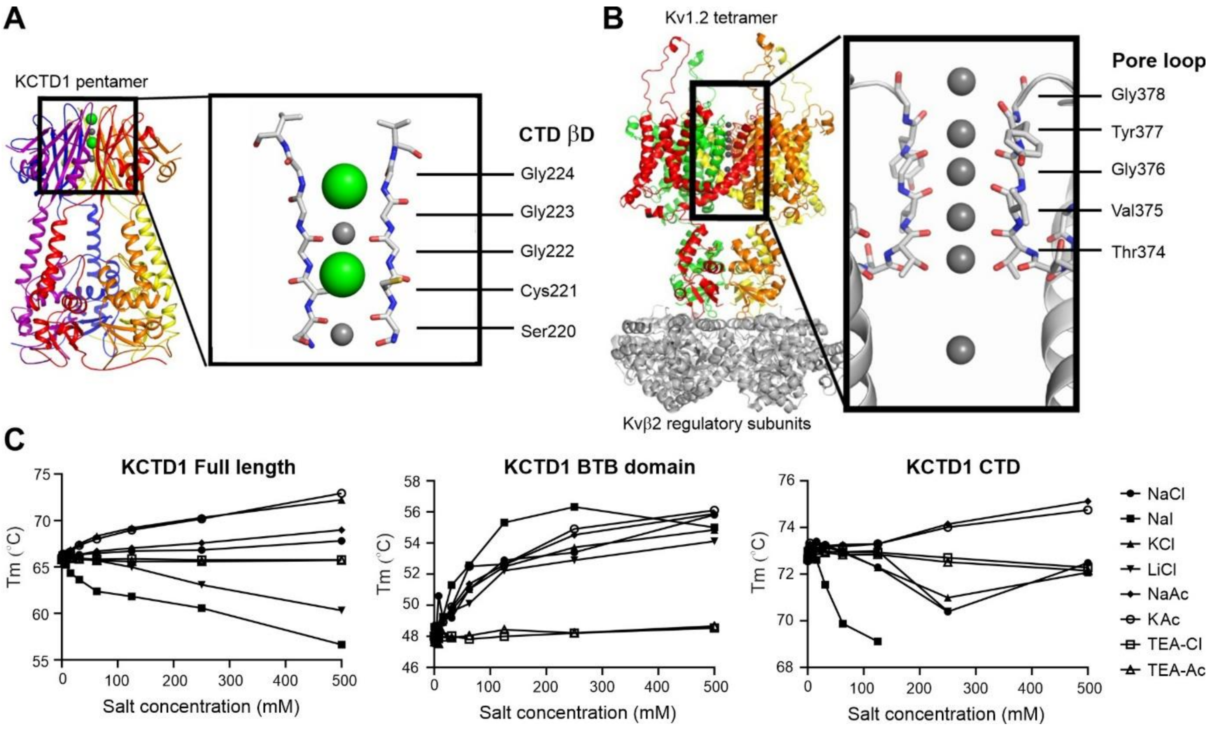

Figure S2. Iodide binding to KCTD1 CTD affects protein stability, Related to Figure 3

(**A**) Ribbon diagram of the pentameric KCTD1 structure. Inset shows a slice through the ion-binding channel (not membrane spanning) in the CTD occupied by two sodium and two iodide ions (gray and green spheres, respectively). The CTD pentamer presents five βD strands that line the channel with their backbone carbonyls. Each strand has a Ser-X-Gly-X-Gly motif that is weakly reminiscent of the Thr-X-Gly-X-Gly motif of pore loop domain (P-domain) selectivity sequence in the Kv channel family. The backbone carbonyls and side chain hydroxyl from the Ser-X-Gly are observed to coordinate the sodium ions. (**B**) Ribbon diagram of the rat Kv1.2 shaker potassium channel (PDB 3LUT). The four subunits of the tetramer are indicated by different colours. The associated Kvβ2 regulatory subunits are coloured gray. Inset shows a slice through the ion pore with potassium ions shown as gray spheres. Residues in the Thr-X-Gly-X-Gly motif of the pore loop domain (P-domain) selectivity sequence are labelled. (**C**) DSF experiments showing apparent melting temperature of full-length KCTD1, as well as the isolated BTB or CTD domains under different salt conditions and concentrations without adjustments for ionic strength. Sodium iodide destabilises the CTD as well as the full-length protein, whereas the BTB domain is stabilized. Protein was buffered in 25 mM HEPES pH 7.5, 0.05% Tween-20 plus indicated salts and SYPRO orange dye. Full length and BTB domain constructs were assayed at 2 µM protein concentration, whereas the KCTD1 CTD construct was assayed at 100 µM protein concentration due to low fluorescent signal. Signal changes for the KCTD1 CTD were also not observable above a concentration of 125 mM NaI.

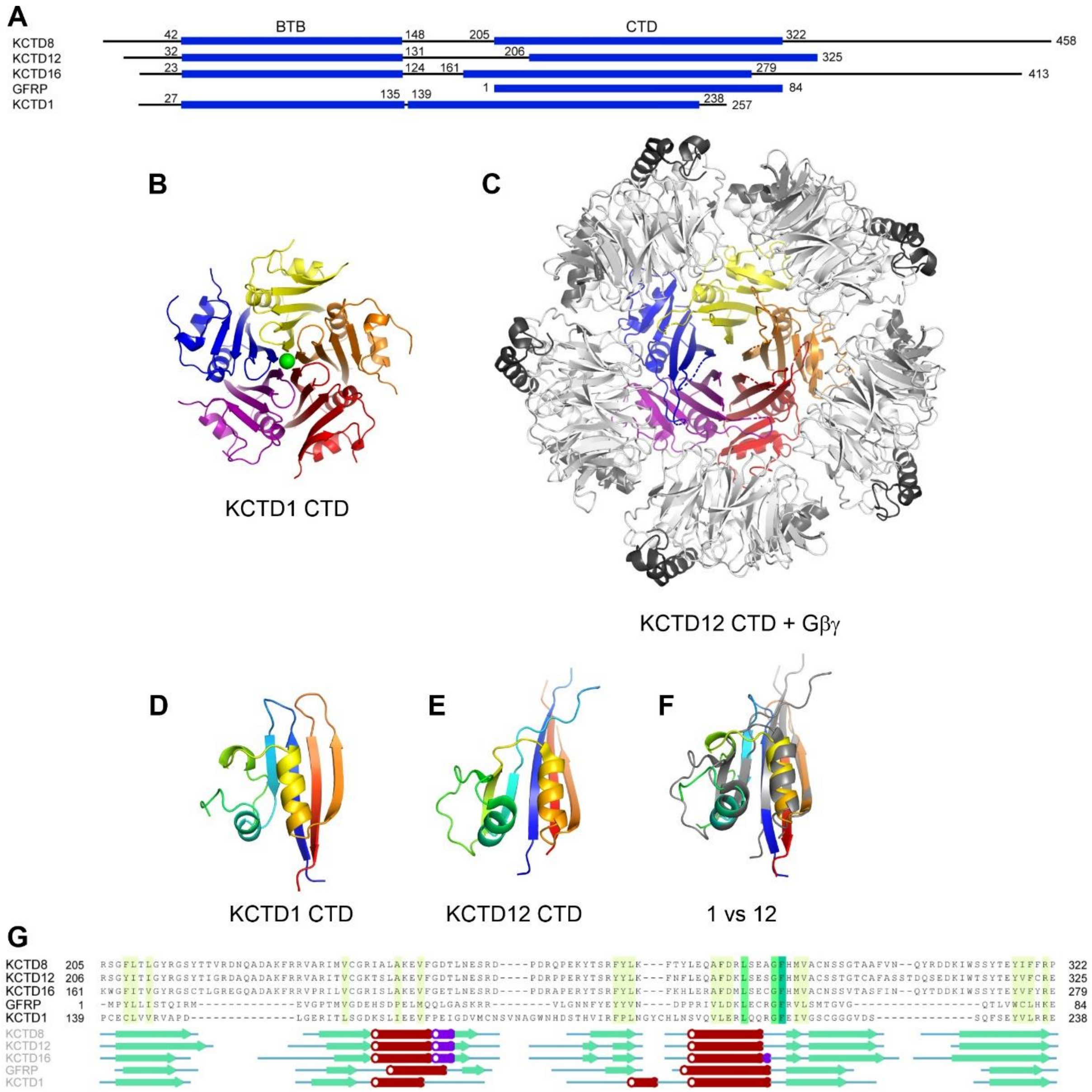

Figure S3. KCTD1 CTD shows structural conservation with KCTD12, Related to Figure 4

(**A**) Domain organisation of KCTD1 and selected homologues. (**B**) KCTD1 CTD pentamer coloured by subunit. Bound iodide ion shown as green sphere. (**C**) KCTD12 CTD pentamer coloured by subunit bound to Gβ (light gray) and Gγ (dark gray) (PDB 6M8S). Subunit topology by rainbow colours for (**D**) KCTD1 CTD and (**E**) KCTD12 CTD. (**F**) Superposition of KCTD1 CTD (rainbow) and KCTD12 CTD (gray) chains. (G) Structure-based sequence alignment and secondary structure elements.

Selected KCTD1 orthologs

Human	MSRPLITRSPASPLNNQ**GIPTP**AQLTKSNAP	31
Dog	MSRPLITRSPASPLNNQ**GIPTP**AQLTKSNAP	31
Chimpanzee	MSRPLITRSPASPLNNQ**GIPTP**AQLTKSNAP	31
Horse	MSRPLITRSPASPLNNQ**GIPTP**AQLTKSNAP	31
Rat	MSRPLITRSPASPLNNQ**GIPTP**AQLTKSNAP	31
Frog	MSRPLITRSPASPLNNQ**GIPTP**AQLTKSNAP	31
Zebrafish	STRPILAHSPVSPLGTA**GIPTP**AQLTKANAP	43
Drosophila	SSAGASSYLHGNHKPIT**GIP**CVAAASRYTAP	136

Selected human KCTD1 paralogs

KCTD1	LNNQ**GIPTP**AQLTKSNAPVHIDV	36
KCTD15	LAAQ**GIP**L**P**AQLTKSNAPVHIDV	62
KCTD8	ASAAAA**P**G**P**CAPSPFPEVVELNV	50
KCTD2	EPGP**G**P**P**ERAGGGGAARWVRLNV	44
KCTD7	ATQA**G**HAL**P**LLPQEFPEVVPLNI	33
KCTD9	PQTDSK**P**PEGLLGFHTDWLTLNV	77
KCTD3	AGGHCGSF**P**AAAAGSGEIVQLNV	24
KCTD20	GNSSVGFGSNSHSQAPEKVTLLV	123
KCTD13	EPGPAAYGLKPLTPNSKYVKLNV	47
KCTD14	QTPLPQSPRPRRPTMSTVVELNV	39
TNFAIP1	GAKPKLSGFKGGGLGNKYVQLNV	34
KCTD5	CSAGLGALAQRPGSVSKWVRLNV	50
KCTD12	GGSGSSSSSAEPPLFPDIVELNV	40

Figure S4. Sequence alignment of pre-BTB regions in KCTD1 orthologs and paralogs, Related to Figure 2

Residues in the N-terminal BTB extension of KCTD1 are shown in bold; Pro20 in human is highlighted in red. These residues are highly conserved in most orthologs, but not in most paralogs, with the exception of KCTD15.
