# Supplementary material for: A BTB extension and ion-binding domain contribute to the pentameric structure and TFAP2A binding of KCTD1
Source: Structure. Author manuscript; Available in PMC 2026 Mar 25. (PMC7618933; doi:10.1016/j.str.2024.07.023)
Supplement: Highlights [file EMS213002-supplement-Highlights.docx]

**Highlights**

- Crystal structure of full-length human KCTD1 that forms a pentamer
- The pre-BTB region is essential for TFAP2A binding and makes domain-swap interactions
- C-terminal domain pentamer forms a central channel with bound sodium and iodide ions
- The structure explains how mutations disrupt KCTD1 in scalp-ear-nipple syndrome
